# Three-Dimensional Imaging of *Drosophila melanogaster*

**DOI:** 10.1371/journal.pone.0000834

**Published:** 2007-09-05

**Authors:** Leeanne McGurk, Harris Morrison, Liam P. Keegan, James Sharpe, Mary A. O'Connell

**Affiliations:** 1 Medical Research Council Human Genetics Unit, Edinburgh, United Kingdom; 2 Systems Biology Program, Centre de Regulació Genòmica, Barcelona, Spain; Fred Hutchinson Cancer Research Center, United States of America

## Abstract

**Background:**

The major hindrance to imaging the intact adult *Drosophila* is that the dark exoskeleton makes it impossible to image through the cuticle. We have overcome this obstacle and describe a method whereby the internal organs of adult *Drosophila* can be imaged in 3D by bleaching and clearing the adult and then imaging using a technique called optical projection tomography (OPT). The data is displayed as 2D optical sections and also in 3D to provide detail on the shape and structure of the adult anatomy.

**Methodology:**

We have used OPT to visualize in 2D and 3D the detailed internal anatomy of the intact adult *Drosophila*. In addition this clearing method used for OPT was tested for imaging with confocal microscopy. Using OPT we have visualized the size and shape of neurodegenerative vacuoles from within the head capsule of flies that suffer from age-related neurodegeneration due to a lack of ADAR mediated RNA-editing. In addition we have visualized *tau-lacZ* expression in 2D and 3D. This shows that the wholemount adult can be stained without any manipulation and that this stain penetrates well as we have mapped the localization pattern with respect to the internal anatomy.

**Conclusion:**

We show for the first time that the intact adult *Drosophila* can be imaged in 3D using OPT, also we show that this method of clearing is also suitable for confocal microscopy to image the brain from within the intact head. The major advantage of this is that organs can be represented in 3D in their natural surroundings. Furthermore optical sections are generated in each of the three planes and are not prone to the technical limitations that are associated with manual sectioning. OPT can be used to dissect mutant phenotypes and to globally map gene expression in both 2D and 3D.

## Introduction

Imaging is a vital tool in all areas of *Drosophila* research; routinely the tissue is dissected and imaged either at low-magnification using the stereomicroscope or at high-magnification using either compound or confocal microscopy. All three techniques are associated with specific disadvantages. Both the stereomicroscope and compound microscope only image in one plane and neither can focus on a point deep within a tissue. Furthermore the upright microscope is used to visualize manually cut sections of tissue, and the processing and sectioning of samples can result in a loss of tissue integrity. Only the confocal can image clearly through the depth of a sample, however it too has disadvantages, for example the tissue may shrink after dissection and can be subjected to shape distortion due to dissection and the mounting procedure. One imaging method that can image internal organs without the need for dissection is optical projection tomography (OPT). OPT images samples in 3D and using associated software the data is used to generate 2D optical sections in each of the three planes, and 3D models. OPT images are estimated to have a pixel resolution of 5–10 µm [1]. However this resolution is dependent upon good signal intensity, a weaker signal will have a resolution that is lower than this estimate.

Since the introduction of OPT there have been several publications of its use in a variety of organisms, for example, the human embryo was imaged and structures within the nervous system were detected without the use of markers [2], [3]. OPT has also been employed to visualize developing plant material [4] and more recently OPT was used to image adult mouse organs [5]. Until now it was believed that the dark exoskeleton of *Drosophila* would prevent the organism from being imaged by OPT. Here we show that not only can the pigment be bleached, but also that adult *Drosophila* is cleared well enough to allow the visualization of anatomical structure in all 3 planes and in 3D. To investigate the benefit of this technique to *Drosophila* research, OPT was used to model neurodegeneneration and to visualize reporter gene expression.

Fly neurodegeneration is regularly visualized using standard histology techniques such as hematoxylin and eosin staining of thin head sections [6], [7]. This technique has many inherent disadvantages, including loss of tissue integrity due to the processing and sectioning procedure, which can create cracks that appear similar to vacuoles in the brain. In addition to this, only one plane can be sectioned, which in flies is often either frontal or horizontal (coronal or transverse). It would therefore be an advantage if a technique could be found which could display the data in all three virtual planes, and in addition, could visualize the adult in 3D. We therefore set out to test OPT for this purpose and to ask whether it could be used as a method for detecting vacuoles in brains of *Adar* mutants that suffer from age-related neurodegeneration due to a lack of RNA editing. The *Adar 5G1* strain contains a deletion over the *Adar* gene [8], which encodes an adenosine deaminase that acts on dsRNA. Once bound to its pre-mRNA substrate ADAR deaminates specific adenosines into inosine, which is read as a guanosine by the translational machinery; this can change amino acid usage thereby increasing protein diversity. Specific editing activity is targeted to transcripts that are expressed in the CNS, some of which encode subunits of ion channels [9], [10]. Editing events can affect splicing of the pre-mRNA [11], and can affect properties of the receptor subunit such as channel permeability [12], and the rate of subunit assembly [13]. Flies lacking *Adar* are ataxic and, with age the flies undergo age-related neurodegeneration [8], [14]. Here we demonstrate that OPT can indeed detect regions of neurodegeneration in whole adult flies and have further confirmed this by subsequent sectioning and staining of the imaged heads.

The efficient bleaching and clearing of *Drosophila* makes it an ideal organism to be imaged by OPT, and may be of use for other microscopy techniques, and so we specifically show the utility of this procedure for confocal microscopy. Additionally OPT may assist in imaging large numbers of *Drosophila* lines that express GFP or β-galactosidase reporters. Large-scale genetic screens have dominated *Drosophila* research for many years. Originally reverse genetics in *Drosophila* relied upon random P-element insertions that induced mutations [15]–[17]. Now mutations can be specifically targeted by homologous recombination [18]–[20] or specific gene products can be depleted by expressing siRNAs to genes in specific tissues or cells [21]–[23]. Cell or neuron specific gene silencing, driven by GAL4, currently underlies the functional dissection of neuronal networks in *Drosophila* and relies upon detailed spatial and temporal expression data on the neuronal GAL4 driver lines. Moreover these expression patterns must be referenced precisely and consistently to specific points in the standard *Drosophila* brain [24], [25].

In order to determine if the reporter gene is expressed in the PNS or CNS the *Drosophila* adult is often bisected or decapitated [26], [27], however in large-scale screens this can be very time consuming. Staining the adult in wholemount would be more efficient, however traditional imaging techniques cannot indicate how well the stain has penetrated and it cannot relate the data to the internal organs. To determine whether OPT is a suitable method to overcome this problem, a β-galactosidase fusion to bovine TAU was imaged [26]. TAU is found within protein aggregations in neurodegenerative diseases, [28], [29] and when it is expressed in *Drosophila* it localizes to the axons in the thorax and CNS [26]. OPT was used to image the β-galactosidase activity in brightfield conditions and these data were then superimposed onto the anatomy of the fly created by the fluorescent signal. The reconstructed data sets in all three planes clearly show that the stain penetrated the fly and was detected within the CNS and PNS.

## Results

### *Drosophila* is suitable for imaging by OPT

**Figure 1:**
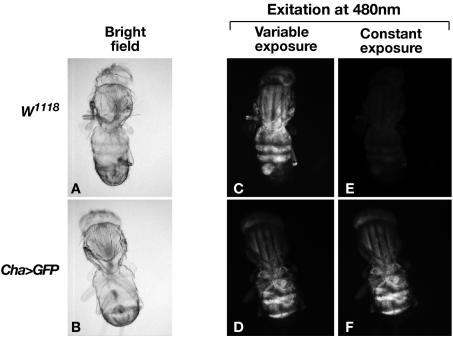
The raw data produced by OPT. *Drosophila* cleared in Murray's clear, is almost transparent when imaged under brightfield conditions (A–B). The cleared fly allows full excitation and emission of fluorescent light when excited at 480±40 nm (C–F). *w^1118^*, and *Cha>GFP* [30] both have detectable levels of autofluorescence (C–D). However when compared at equal exposure times the GFP line shows greater fluorescence (E–F).

Previously it was thought that the pigment of the *Drosophila* exoskeleton would be too dark to allow full transmission of light and so initially it was essential to ascertain whether *Drosophila* was a suitable organism for OPT. The *Drosophila* adult was fixed in paraformaldehyde, and then the pigment was bleached in hydrogen peroxide, before being dehydrated and cleared as previously described for vertebrate embryos [1]. The *Drosophila* adult was successfully cleared to almost transparent levels when bathed in 1 part benzyl alcohol and 2 parts benzyl benzoate (Murray's clear) and hence this treatment results in full transmission of white and fluorescent light (Figure 1 and Movie S1). Upon excitation by light of specific wavelengths some tissues auto-fluoresce. In OPT this is a useful tool as it can provide information on shape and structure without staining for cellular markers, and can indeed provide anatomy onto which gene expression patterns can be mapped [1], [3]. We wanted to determine whether this technique could be used to visualize the internal anatomy of the intact *Drosophila*. The wild-type fly-line *w^1118^* was fixed in paraformaldehyde, a fixative known to create autofluorescence, and was found to have sufficient autofluorescence to mark out internal anatomy when excited at 480 nm (Figure 1C and E). To determine whether there was increased fluorescence in a GFP background a fly expressing GFP in the cholinergic neurons (*w;* C*ha-GAL4 (19B), UAS-GFP S65T*) [30] was imaged by OPT and compared to *w^1118^*. When the two fly strains, comprising of six flies in total, were imaged at equal exposure times it was seen that two out of three *Cha>GFP* flies had significantly more fluorescence not only in the CNS but also throughout the body (Figure 1E and F). Exposure time was set at a level that was just below saturation levels. It should be noted there is a difference in fluorescence levels between flies with the same background and how efficiently the fly has been bleached can influence this.

### Visualizing the data in 2D and 3D

**Figure 2:**
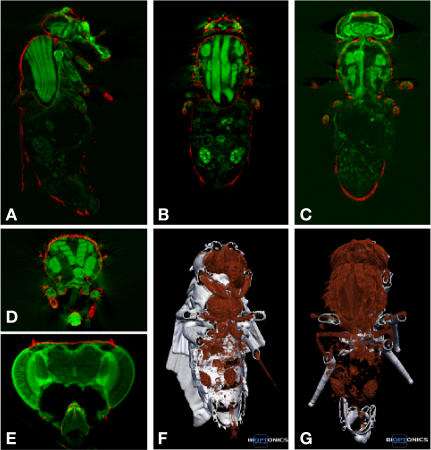
The data output. The data from the scans are reconstructed in 3D, the two datasets, brightfield (red, exoskeleton) and fluorescent (green, anatomy), are superimposed, and the information is displayed in all three planes (A–D). Single heads can be imaged (E). The data can also be displayed and explored in 3D (F, G).

The data were visualized in 2D in each of the three planes and the two datasets, fluorescent and brightfield, were superimposed (Figure 2). The brightfield data mark out the fly exoskeleton, and are colored red. Due to the clearing of the fly the exoskeleton is almost transparent and in some regions it is completely transparent resulting in some optical sections lacking the brightfield signal in some regions (Figure 2E). The fluorescent image, in green, clearly delineates various anatomical features such as the thoracic muscles, the heart and the ovaries, and to some degree the gut (Figure 2A–D and Movie S2). The variation in signal intensity in the fly means that the a reasonable threshold must be applied such that there is not over saturation of strong signal, for example the thorax, and loss of weaker signal such as the gut. Importantly, as shown in Figure 2F and Movie S3, we were able to visualize the adult anatomy in 3D. To test OPT at its maximum resolution, individual *Cha>GFP* heads were imaged (Figure 2E). The brain can be seen clearly, and regions such as the mushroom body calyces and retina are easily identifiable.

### Clearing the *Drosophila* head for use for confocal microscopy

**Figure 3:**
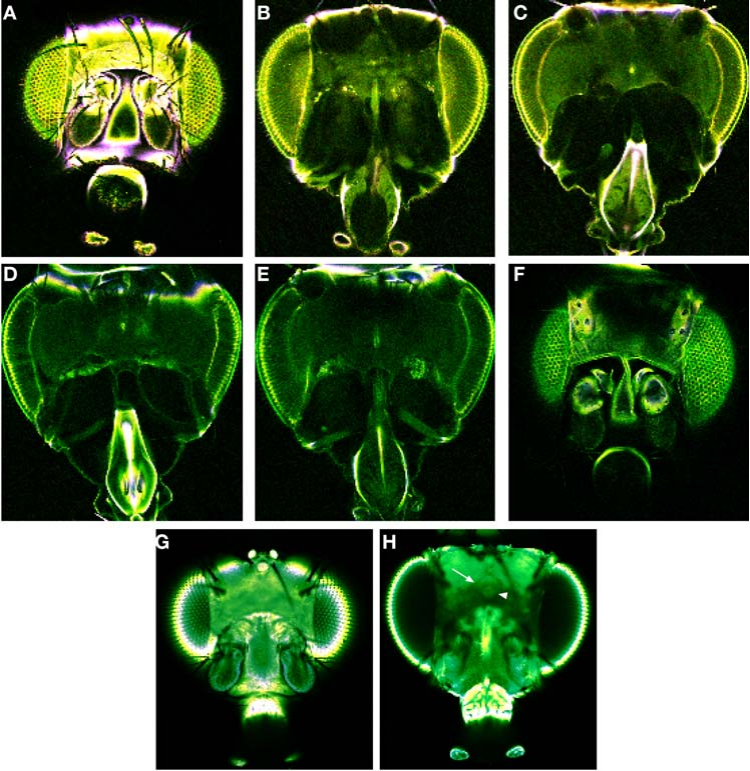
Visualizing through the head cuticle with confocal microscopy. The CNS can be visualized through the cleared head. Both *w^1118^* (A–F) and *Cha>GFP* (G–H) were visualized. The brain was imaged from the front (A–C, and G–H) and back (D–F) and structures such as the fan shaped body and ellipsoid body were detected (H arrow and arrowhead).

We have shown that OPT can be used to image the structure within the intact head after efficient bleaching and clearing. This method may also assist confocal microscopy, where at present dissection is necessary as the exoskelton prevents visualization of the CNS. To test this clearing method for confocal microscopy heads of *Cha*>*GFP* and *w^1118^* were fixed and cleared. Autofluorescence has been used to visualize neuroanatomical structure in wax sections of the *Drosophila* brain [31] and so assessment of the clearing procedure and its use in confocal microscopy relied upon autofluorescence. Clearing was efficient enough to allow visualization of the CNS (Figure 3A–H), and internal structures such as the fan shaped body and the ellipsoid body were detected (Figure 3H). One main advantage of OPT is that the data is imaged from 400 angles, so if a structure is blocked by a pigmented area at one angle it can be imaged from another angle. This is not the case for confocal microscopy, imaging occurs in only one plane and so any data underneath a pigmented region is lost (Figure 3G–H). However this method does opens up the possibility that the whole head may be used in immunohistochemistry.

### Neurodegeneration is detected by OPT

**Figure 4:**
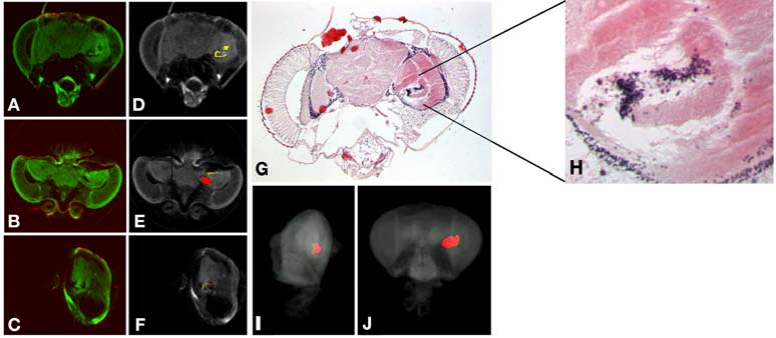
Identification of neurodegenerative vacuoles using OPT. OPT was used to identify regions of neurodegeneration in the brains of flies lacking the RNA editing enzyme *Adar*. Regions that lacked fluorescence were identified from sections in different orientations (A–C) and then these potential vacuoles were highlighted as domains (with different colors) using the MAPaint software (D–F). This was repeated in all sections that the vacuole extended into and this was reconstructed in 3D with respect to the *Drosophila* head. (I–J). The vacuoles were confirmed by haematoxylin and eosin staining of the OPT imaged head (G–H).

OPT was explored as a method to assess vacuolization in the brain of *Adar* mutants. *Adar 5G1* males, in the *Cha>GFP* background (*w; Cha-GAL4 (19B)*, *UAS-GFP S65T*), were aged until 20 days and visualized using OPT (Figure 4A–C). Regions that lacked GFP signal were marked as regions of possible neurodegeneration. MAPaint software, developed by the Edinburgh Mouse Atlas Project [32]–[34], was used to analyze these vacuoles. The regions that lacked fluorescence were painted and this was repeated for each section that showed putative vacuoles (Figure 4D–F). The painted regions (domains) were then processed into 3D with respect to the *Drosophila* head (Figure 4I and J and Movie S4). In order to determine whether this painted domain was a region of neurodegeneration the heads were removed from the agarose and subsequently embedded in paraffin wax. Frontal sections were cut and sections that contained regions of neurodegeneration were compared to the OPT sections. The paraffin sections confirmed that vacuolization of the optic lobe had indeed occurred (Figure 4G and H). This confirms that OPT can be used to visualize neurodegeneration from within the intact adult head, which is a procedure that traditionally relies upon analysis of heavily processed paraffin sections.

### β-galactosidase staining can be mapped onto an anatomical atlas of *Drosophila*

**Figure 5:**
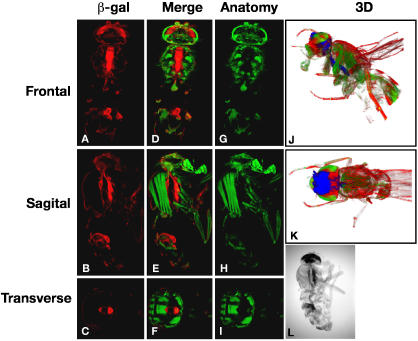
Visualization of â-galactosidase activity. The *tau-lacZ* enhancer trap flies [26] were stained for â-galactosidase and imaged in brightfield (L). The brightfield channel captured both the â-galactosidase activity and the transparent exoskeleton, both of which are represented in red. The brightfield signal was then superimposed onto the anatomical information generated from the autofluorescence of the same specimen, this is shown in green (A–I). Finally the β-galactosidase activity was painted blue, and reconstructed in 3D along with the other two channels, brightfield red and anatomy green (J–K).

Wholemount β-galactosidase staining of a bisected adult fly expressing a TAU-LacZ fusion revealed that TAU, a microtubule binding protein, localizes to the axons of the thoracic ganglion and adult CNS [26]. However, when imaging using a standard stereomicroscope only surface staining can be detected. Therefore one advantage of this technique is that one is able to determine how penetrant the stain is without any manipulation. Staining for β-galactosidase activity was carried out on wholemount adults and the staining pattern was imaged in the brightfield channel using OPT (Figure 5A–C and L). The brightfield data were superimposed onto the anatomy that was obtained from the fluorescent channel. The β-galactosidase activity was clearly seen to be in the region of thoracic ganglion (Figure 5D–I and Movie S5). This staining is distinct from the gut that has endogenous β-galactosidase activity in *Drosophila*. Finally the 3D reconstruction of the data clearly shows that the staining detected in the brain does indeed extend along the ventral nerve cord and connect to the thoracic ganglion (Figure 5J–K and Movie S6).

## Discussion

OPT is an imaging technique that models data in 3D. Originally it was designed to image the mouse embryo [1] but it has since been used to image human embryos, adult mouse tissue and plant tissue [2], [4], [5]. This is the first time that imaging of the *Drosophila* adult in 3D has been reported. We show that *Drosophila* can be cleared and that the clearing permits the transmission of white and fluorescent light to allow detection of detailed anatomy. Upon excitation with fluorescent light, the cleared adult emits light, even from deep within the intact body, so that detailed 3D images of the *Drosophila* anatomy can be produced. The clearing of the *Drosophila* adult is very efficient and we also show that it can be used when imaging *Drosophila* using confocal microscopy. Here we show that OPT can be applied to two important areas of *Drosophila* research, analysis of mutant phenotype, namely neurodegeneration, and 3D visualization of reporter gene expression.

*Drosophila* is widely accepted as an important model organism for studying neurodegenerative diseases [35], [36]. Previously identification of neurodegeneration in *Drosophila* has relied upon sectioning of wax embedded heads [6], [8], however the difficulties associated with sectioning often result in damage to the tissue, which can be misinterpreted as neurodegenerative vacuoles. Here we have shown that OPT can be used to visualize neurodegeneration in 3D from within the intact adult *Drosophila* head and have confirmed by sectioning that indeed these brains contained vacuoles. It is possible to warp high-resolution data captured from wax sections onto the framework obtained by OPT and display it in 3D [2], [3], [32]–[34].

As well as analyses of mutant phenotype, gene expression patterns can provide insight into gene function. Large-scale screens to identify expression patterns of interest commonly use *GFP* or *LacZ* reporter genes [37]. Currently great effort is directed toward elucidating the link between neuronal networks and neuronal function. The use of directed mutagenesis strategies and the creation of network specific GAL4 drivers will be fundamental to this field. However the correct detailing of GAL4 expression patterns is vital, and OPT has the potential to benefit this in two ways. Firstly we show the *Drosophila* can be stained in wholemount and this could aid rapid identification of drivers that are specific to the CNS or the PNS. Secondly, it images the brain from within the intact head capsule and can therefore potentially provide a structure which could aid the construction of a standard atlas that represents the true size and shape of the adult *Drosophila* brain. We have also shown that this processing procedure can be used to image with the confocal the brain from within the head capsule. This method could also be used to create a reference brain from mapping gene expression patterns and can give detailed images that show neuroanatomical structures such as the fan shaped body and ellipsoid body.

This is the first report of imaging through the *Drosophila* cuticle in 3D. At the current level of resolution OPT provides detailed images on the gross anatomical structure of the fly. The anatomy shown here is dependent upon autofluorescence and this varies between flies and within the fly itself, therefore the user must set a threshold of intensity which is optimum for each fly. This may result in the loss of signal from gut structure in the abdomen, but other structures such as the nervous system, cardia, thorax muscles and gonads are easily visible. These images can be used as an anatomical framework onto which gene expression patterns can be mapped, as demonstrated here with *tau-lacZ*. Furthermore the data has the potential to be used as a framework onto which high-resolution data can be superimposed allowing it to be displayed in 3D [2], [3], [32]–[34]. By assisting many of the traditional image capture methods such as compound and confocal microscopy OPT may potentially benefit all areas of *Drosophila* research.

## Materials and Methods

### Fly stocks and fly maintenance

All fly stocks were raised on standard corn meal-agar medium supplemented with live baker's yeast. For aging experiments flies were maintained at 25°C on standard corn meal-agar medium but the vials were not supplemented with live bakers yeast. A single fly was maintained in a vial and each vial was tipped on every 1–3 days. Prof. Paul Salvaterra, Stanford University provided *w; Cha-GAL4 (19B)*, *UAS-GFP S65T* and the *tau-lacZ* enhancer trap line, 3,358, was obtained from Prof J. Thomas at the Salk Institute [26].

### Sample preparation and imaging for OPT

Whole flies were fixed in 4% paraformaldehyde for 8 hours whereas heads were fixed for 4 hours. The samples were then bleached in hydrogen peroxide and paraformaldehyde at 4°C. Samples were mounted in 1% agarose, dehydrated in methanol and then cleared in BABB (1 part Benzyl Alcohol: 2 parts Benzyl Benzoate). The sample was imaged in both the brightfield and fluorescence channels (480 nm) and the images were reconstructed using in-house software designed as part of the Edinburgh mouse atlas project [33], [34]. Bioptonics 3001 OPT Scanner software was used to generate the 3D adult flies. Neurodegeneration was mapped using the MAPaint programme also designed as part of the Edinburgh mouse atlas project [33], [34].

### Imaging *Drosophila* heads using the confocal microscope

Heads were fixed in 4% paraformaldehyde for 4 hours at room temperature, after an overnight dehydration step in methanol they were cleared in BABB for at least six hours. The heads were mounted with a raised coverslip in a small amount of BABB. To achieve maximum fluorescence heads were visualized using the following emission filters: LP650 BP500-530 BP 565-615.

### Removal of heads from agarose and embedding into paraffin wax

Agarose was removed from the sample by incubation in warm 0.29M sucrose. The sample was dehydrated, embedded in paraffin wax and 7 µm sections were stained with haematoxylin and eosin

### β-glactosidase staining of *tau-lacZ* expressing flies

Whole *tau-lacZ* adult flies were fixed for 3 hours in 4% paraformaldehyde at 4°C, and rinsed for one hour. Flies were incubated in reaction buffer (pH 7.2) containing 1 mg/ml 5-bromo-4-chloro-3 indolyl 3-D-galactoside (X-GAL) for 12–18 hours at 37°C with rotation. Flies were then bleached in 15% hydrogen peroxide and 2% paraformaldehyde at 4°C for 2–3 days and then mounted for OPT.

## Supporting Information

Movie S1 An example of raw data produced under white light. Four hundred images are captured as the cleared adult rotates a full 360°.
(1.38 MB AVI)

Movie S2 Optical sections through the frontal plane of the adult. The fluorescent channel (green) delineates the anatomy, and the brightfield channel (red) marks out the exoskeleton.
(3.05 MB MPG)

Movie S3 3D reconstruction of the intact *Drosophila* adult. The exoskeleton from the brightfield channel is gray and the anatomy from the fluorescent channel is brown.
(16.15 MB AVI)

Movie S4 Reconstruction of the vacuolization in Adar mutant brains. The vacuolization was marked (painted) on each of the optical sections; the painted regions were then saved as a domain and superimposed onto the fluorescent *Drosophila* head.
(1.54 MB MPG)

Movie S5 Optical sections through a tau-lacZ adult stained for β-galactosidase activity. The fluorescent signal (green) identifies the adult anatomy and the brightfield signal (red) marks out the β-galactosidase activity and the exoskeleton.
(3.04 MB MOV)

Movie S6 3D reconstruction of the β-galactosidase activity of a tau-lacZ expressing adult. The β-galactosidase activity was painted and saved as a separate domain. This along with the brightfield signal (red) and fluorescent signal (green) were reconstructed in 3D using Bioptonics 3001 OPT scanner software.
(12.11 MB MOV)
